# Supplementary material for: COZOID: contact zone identifier for visual analysis of protein-protein interactions
Source: BMC Bioinformatics. 2018 Apr 6;19:125. doi: 10.1186/s12859-018-2113-6 (PMC5889581; doi:10.1186/s12859-018-2113-6)
Supplement: Supplementary file 2 — Software build. Executable binary file of the software tool. (ZIP 118,784 kb) [file 12859_2018_2113_MOESM2_ESM.zip › cozoid/platform/docs/testng-6.5.1-javadoc.zip!/org/testng/annotations/DataProvider.html]

DataProvider (TestNG)


---


|  |  |  |
| --- | --- | --- |
| **Overview** \| **Package** \| **Class** \| **Tree** \| **Deprecated** \| **Index** \| **Help** | |  |
| **PREV CLASS**   **NEXT CLASS** | **FRAMES**    **NO FRAMES**     **All Classes** |  |
| SUMMARY: REQUIRED \| OPTIONAL | DETAIL: ELEMENT |  |


---


## org.testng.annotations Annotation Type DataProvider

---

``` @Retention(value=RUNTIME) @Target(value=METHOD) public @interface DataProvider ```

Mark a method as supplying data for a test method. The data provider name
defaults to method name.
The annotated method must return an Object[][] where each
Object[] can be assigned the parameter list of the test method.
The @Test method that wants to receive data from this DataProvider
needs to use a dataProvider name equals to the name of this annotation.

**Author:**
:   cbeust

---

| **Optional Element Summary** | |
| --- | --- |
| `int[]` | `indices`             Which indices to run from this data provider, default: all. |
| `java.lang.String` | `name`             The name of this DataProvider. |
| `boolean` | `parallel`             Whether this data provider should be run in parallel. |

### name

```
public abstract java.lang.String name
```

:   The name of this DataProvider.

    :   **Default:**: ""

---


### parallel

```
public abstract boolean parallel
```

:   Whether this data provider should be run in parallel.

    :   **Default:**: false

---


### indices

```
public abstract int[] indices
```

:   Which indices to run from this data provider, default: all.

    :   **Default:**: {}


---


|  |  |  |
| --- | --- | --- |
| **Overview** \| **Package** \| **Class** \| **Tree** \| **Deprecated** \| **Index** \| **Help** | |  |
| **PREV CLASS**   **NEXT CLASS** | **FRAMES**    **NO FRAMES**     **All Classes** |  |
| SUMMARY: REQUIRED \| OPTIONAL | DETAIL: ELEMENT |  |


---
